# Attenuated activation of the anterior rostral medial prefrontal cortex on self-relevant social reward processing in individuals with autism spectrum disorder

**DOI:** 10.1016/j.nicl.2020.102249

**Published:** 2020-03-19

**Authors:** Motofumi Sumiya, Yuko Okamoto, Takahiko Koike, Tsubasa Tanigawa, Hidehiko Okazawa, Hirotaka Kosaka, Norihiro Sadato

**Affiliations:** a Division of Cerebral Integration, National Institute for Physiological Sciences, Aichi, Japan; b Research Fellow of the Japan Society for the Promotion of Science, Tokyo, Japan; c Graduate School of Informatics, Nagoya University, Aichi, Japan; d Research Center for Child Mental Development, University of Fukui, Fukui, Japan; e ATR-Promotions, Brain Activity Imaging Center, Kyoto, Japan; f Advanced Telecommunications Research Institute International, Kyoto, Japan; g Biomedical Imaging Research Center, University of Fukui, Fukui, Japan; h Department of Neuropsychiatry, University of Fukui, Fukui, Japan

**Keywords:** Autism spectrum disorder, Functional magnetic resonance imaging, Reward processing, Self, Medial Prefrontal cortex

## Abstract

The social motivation hypothesis posits that people with autism spectrum disorder (ASD) find social stimuli less rewarding and are therefore less motivated towards social interaction than people with neuro-typical development (TD). However, the less rewarding social stimuli characteristics during social interaction for people with ASD are largely unknown. The contingent positive responsiveness of others relevant to self-action motivates the early development of social interaction, thus representing a social reward. As individuals with ASD often exhibit atypical responses to self-relevant stimuli in their early life, we hypothesized that the self-relevant responses of others are less rewarding for individuals with ASD. To test this hypothesis, we conducted a functional magnetic resonance imaging study using a social contingency task. During the task, the participants attempted to make the audience laugh by telling funny jokes and thus activating the anterior rostral medial prefrontal cortex (arMPFC) of TD individuals (Sumiya et al., 2017). We explicitly predicted that the atypical activation of the arMPFC is related to the reduced reward value of self-relevant responses to others in individuals with ASD. Thirty-one adults with ASD and 24 age- and intelligence quotient-matched TD adults participated in the study. Participants with ASD reported significantly lower pleasure after the audience's responses to their own actions than those in the TD group. Correspondingly, the self-related activation of the arMPFC, defined by the results of our previous study, was attenuated in the ASD group compared to the TD group. The present findings indicate that weak self-relevant outcome processing mediated by the arMPFC of individuals with ASD dampens the rewarding nature of social interaction.

## Highlights

• Social reward characteristics for people with ASD are unknown.
• Social contingency motivates social interaction and consequent rewards.
• Social contingency tasks are known to activate the arMPFC.
• The arMPFC response in ASD patients during a social contingency task was attenuated.
• Weak responses to social contingency in ASD reduce social interaction rewards.

## 1 Introduction

Autism spectrum disorder (ASD) is a neurodevelopmental disorder characterized by difficulties in social communication and social interaction as well as restricted, repetitive patterns of behavior, interests, or activities (Diagnostic and Statistical Manual of Mental Disorders, Fifth Edition [DSM-5]; American Psychiatric Association, 2013). Recently, a theoretical account of ASD, called social motivation theory, suggests that autistic symptomatology and social impairments might be related to deficits in social reward processing (Chevallier et al., 2012). This framework assumes that deficits in social cognition are preceded by and secondary to diminished social motivation. A recent systematic review about behavior and imaging studies on this hypothesis (Bottini, 2018) and a meta-analysis of imaging studies (Clements et al., 2018) revealed contradictory findings. For example, Bottini (2018) reviewed 27 studies, 15 of which found evidence of the social motivation hypothesis whereas 12 studies found contradictory evidence, likely caused by the different methodologic approaches used (e.g., experimental paradigm or stimuli topography). Due to the contradictory findings, Bottini (2018) raised the question of social reward specificity. Many previous studies targeting reward processing in people with ASD focused on extrinsic rewards (face, money, food, etc.) with a non-interactive paradigm (e.g. Delmonte et al., 2012; Dichter et al., 2012). Bottini (2018) concluded that specifying reward processing, including the reward type and experimental paradigm, is critical for testing the social motivation hypothesis.

In this context, the definition of social knowledge in general, including social rewards, is crucial. Lewis (1999) argued that "the definition of social knowledge involves the relationship between the knower and the known rather than characteristics of people as objects." "If the self is not involved, then the people are being treated as objects: when the self is involved, people are being treated as people." From this perspective, previous studies of extrinsic rewards dealing with the perceptual component are lacking self-involvement.

The contingency between self-action and other's responses (Jones and Gerard, 1967) is an example of self-involvement that makes the resultant responses of others rewarding. Behavioral contingency is a central construct in many theories of early development (Lewis and Goldberg, 1969; Schaffer, 1984; Symons and Moran, 1994). Contingent adult responsiveness is considered to have a positive effect on an infant, while non-contingent stimulation is thought to have negative consequences by reducing an infant's motivation to participate in contingency relationships and impairing an infant's ability to detect contingent relationships (Dunham et al., 1989). Also, early developmental studies on infants reported the expression of a positive effect accompanied by the contingency of other's responses to self-action (Kasari et al., 1990; Mundy et al., 1992). Recently, a functional magnetic resonance imaging (fMRI) study by Warnell et al. (2018) showed that engaging in social interaction recruits the reward system; thus, social interaction is rewarding. These studies indicate that contingent positive responses relevant to self-action are social rewards and emphasize the role of self-relevant information processing as a powerful foundation for developing social motivation (Grossmann, 2015; Mundy, 2018).

Nevertheless, such self-relevant processing is atypical in individuals with ASD from an early age to adulthood (for review: Cañigueral and Hamilton, 2019; Huang et al., 2017; Nijhof and Bird, 2019; Perrykkad and Hohwy, 2019; Uddin, 2011; Williams, 2010). For example, the lack of orienting to one's own name is a common warning sign for children who are later identified as having ASD (Leekam and Ramsden, 2006; Mars et al., 1998; Nadig et al., 2007; Osterling and Dawson, 1994; Zwaigenbaum et al., 2005). Also, youths and adults with ASD demonstrate reduced attention and memory for self-relevant objects compared with neuro-typical developing (TD) individuals (Grisdale et al., 2014; Henderson et al., 2009). These studies imply that individuals with ASD also have hindered self-relevant processing in an interactive environment; the difficulty in processing self-relevant information attenuates the reward value of social interaction.

Recently, we found the importance of self-relevant stimuli processing for enjoying social interaction in TD adults (Sumiya et al., 2017). We conducted an fMRI study on TD adults with a social contingency task in which the participant attempted to make the audience laugh by telling funny jokes. The findings show that the response of others, laughter, that was contingent upon the participants’ actions, activated the reward system; thus, social interaction can induce rewards. Also, the self-related activation of the anterior rostral medial prefrontal cortex (arMPFC; Brodmann Area 10) modulated the effective connectivity from the auditory cortex to the ventral striatum. Hence, the arMPFC has a gating function regarding sensory input associated with the responses of others during value processing. This study indicated that arMPFC activation associated with self-relevant outcome processing is important to enjoy social interaction. Therefore, we hypothesized that the reduced motivation for social interaction in people with ASD may be caused by arMPFC hypo-function associated with the processing of the responses of others as self-relevant outcomes during social interaction.

## 2 Materials and methods

### 2.1 Participants

Thirty-one adults with ASD (4 females; age, mean ± standard deviation: 29.1 ± 7.8 years) and 24 young male TD adults (age: 28.96 ± 7.07 years) participated in this study (Table 1). TD participants were newly recruited for this study and this group did not overlap with controls in our previous report (Sumiya et al., 2017). The control group did not include females as the majority of the ASD group was male, and since our previous study (Sumiya et al., 2017) showed no gender difference in behavior or neural response. ASD participants were recruited from the outpatient department of the University of Fukui Hospital and diagnosed by a psychiatrist (H.K) based on the DSM-5 diagnostic criteria (American Psychiatric Association, 2013). To establish a DSM-5 diagnosis, H.K. applied the Diagnostic Interview for Social and Communication Disorders, designed to collect information about various developmental and behavioral features including social functioning and communication (Wing et al., 2002). TD individuals were recruited from the local community. Participants of both groups were excluded if they had a history of major medical or neurological illnesses including epilepsy, significant head trauma, or a lifetime history of alcohol or drug dependency.

**Table 1:** Demographic data.

	TD group			ASD group			p value
Number	24			31			
Age	28.96	±	7.07	29.13	±	7.81	0.934
FSIQ	112.54	±	7.71	107.67	±	12.29	0.096
AQ total	15.25	±	5.81	33.6	±	5.71	< 0.001

Intelligence quotient (IQ) scores were obtained using the Wechsler Adult Intelligence Scale-III (Wechsler, 1997) and individual autistic traits were measured via the Autism Spectrum Quotient (AQ) score (Baron-Cohen et al., 2001) from all participants except 1 ASD participant without an AQ score and 1 ASD participant without an IQ score, who declined the assessment. There were no group differences in age and full-scale IQ (FSIQ) (all *p* > 0.05) values and the FSIQ scores of all participants were > 80 (Table 1). The total AQ and Social Responsiveness Scale scores were significantly higher in individuals with ASD than in TD individuals (all *p* < 0.001, independent-sample *t*-test) (Table 1).

All participants provided written informed consent. The study was approved by the ethical committee of the University of Fukui (Japan). All methods were carried out in accordance with the approved guidelines.

TD, neuro-typically developing; ASD, autism spectrum disorder; Number, number of participants; FSIQ, full-scale intellectual quotient assessed by the Wechsler Adult Intelligence Scale, Third Edition (Wechsler, 1997); AQ, autism spectrum quotient (Baron-Cohen et al., 2001). Age, FSIQ, and AQ scores are shown as the mean ± the standard deviation. The p values indicate the results of independent sample t-tests comparing the ASD and TD groups.

### 2.2 Experimental design

Twenty participants with ASD and all TD participants completed 2 tasks: the pseudo-interactive joke task in the MRI scanner and the supplementary luminance task outside the MRI room. The luminance task was conducted after the pseudo-interactive joke task and was used to confirm the participant's ability to discriminate abstract objects by comparing them with their own criteria. The other eleven participants with ASD, who didn't want to go into the MRI scanner, completed the pseudo-interactive joke task in the experimental room.

#### 2.2.1 Pseudo-interactive joke task

In this task, 1 of the 2 actors (SELF or Computer [PC]) uttered a joke (speaker), and a listener made a response after the utterance. There were 3 listener responses (Group laughter, Single laughter, and No laughter). Accordingly, this task contained 6 conditions: SELF_Group (i.e., the self-utterance of a joke followed by group laughter), SELF_Single, SELF_No, PC_Group, PC_Single, and PC_No.

##### 2.2.1.1 Stimuli

The 90 funniest jokes were categorized into 6 sets (15 jokes in each set) such that the mean rating of funniness was matched between them. Each set was pseudo-randomly chosen for each task condition. For details regarding joke selection and auditory stimuli preparation, see the methods section of our previous study (Sumiya et al., 2017).

##### 2.2.1.2 Stimulus presentation

In the MRI experiment, visual stimuli presentation and response collection were conducted with Presentation software (Neurobehavioral Systems, Berkeley, CA, USA) implemented on a Windows-based desktop computer. Visual stimuli were presented on a screen by a liquid-crystal display projector. Participants viewed the visual stimuli via a mirror attached to the head coil of the MRI scanner. Participants listened to auditory stimuli through MRI-compatible headphones (Visual Stim Controller; Resonance Technology Inc., CA, USA). Participants’ utterances were recorded with an opto-microphone system (Optoacoustics Ltd., Moshav Mazor, Israel). Behavioral responses were collected via an optical button box (HHSC-1 × 4; Current Designs Inc., Philadelphia, PA, USA).

For the luminance task, the visual stimuli presentation and response collection were conducted with Presentation software (Neurobehavioral Systems, Berkeley, CA, USA) implemented on a Windows-based laptop computer.

##### 2.2.1.3 Cover story

Participants were instructed to read the punchline of jokes aloud in 1 condition and asked to listen to the punchline of jokes played by a computer in the other condition (PC condition). Participants were encouraged to read the punchline in a funny way. Before the experiment, participants met an individual whose sex was the same as their own; they were informed that this individual would be listening to the jokes in another room and evaluating the funniness of the jokes by pressing buttons corresponding to 1 of the 3 auditory responses. The participants were told that this listener was different from the reader of the joke in the PC condition. Although the listener's response was pre-determined (as described in the section on stimuli), participants were told that the listener evaluated the funniness of the joke. All participants confirmed their belief that another real person evaluated the uttered jokes.

##### 2.2.1.4 Task schedule

**Fig. 1:**
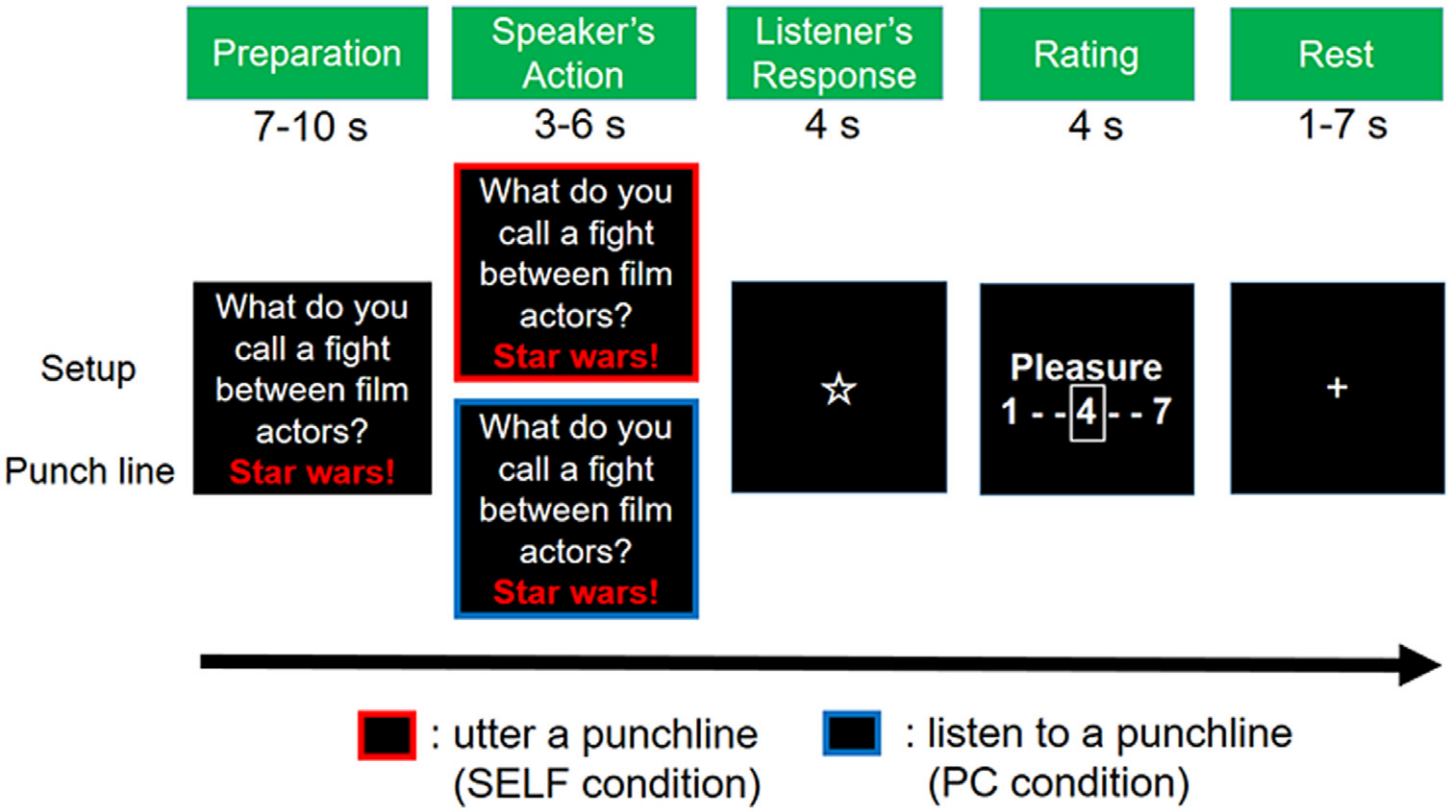
Task sequence.

Participants conducted 3 runs, each of which lasted for 800 s. Each run consisted of 30 trials lasting for 25 s (750 s). Each of the 6 conditions was presented 5 times in each run. A 25-s baseline was inserted before the first trial and after the last trial (750 + 50 = 800 s). Each trial consisted of 5 phases: Preparation, Speaker's Action, Listener's Response, Rating, and Rest (Fig. 1).

Each trial consisted of 5 phases: Preparation, Speaker's Action, Listener's Response, Rating, and Rest. In the Preparation phase, the participant observed and listened to the setup of a joke. Two conditions were prepared in the Action phase: when the screen frame turned red, the participant uttered the punchline of the joke (SELF condition) and when the screen frame turned blue, the participant listened to the punchline read aloud by the PC (PC condition). Each punchline was new and presented only once. In the Response phase, the participant heard 1 of 3 responses from the listener: laughter of people (Group laughter), laughter of a single individual (Single laughter), or silence (No laughter). The participant then rated his or her pleasantness by pressing buttons in the rating phase.

###### 2.2.1.4.1 Preparation phase

The setup and punchline of a joke were visually presented on the screen. Four seconds after the joke appeared the setup was read aloud in an experimenter's voice (the same voice as in the PC condition). This phase took between 7 and 10 s, depending on the length of the joke.

###### 2.2.1.4.2 Speaker's action phase

One of the two frame colors was superimposed on the visual stimuli. When a red frame appeared, the participant was asked to read the punchline aloud (SELF condition). Conversely, when a blue frame was presented, the participant was asked to listen to the punchline that was read aloud by the PC (PC condition). This phase took 3 s–6 s depending on the length of the joke.

###### 2.2.1.4.3 Listener's response phase

One of the three levels of laughter was presented while a star mark was visually presented for 4 s (Group laughter/Single laughter/No laughter). In the Group or Single laughter conditions, participants heard 3.3 s of laughter 0.5 s after the star mark appeared. No sound was presented in the No laughter condition.

###### 2.2.1.4.4 Rating phase

Participants reported the degree of subjective pleasure using a 7-point Likert scale (1 = no pleasure, 7 = very pleasurable). Participants pressed 2 buttons with their right index and middle fingers to choose their subjective pleasure rating. The initial position of choice was pseudo-randomized on the rating scale.

###### 2.2.1.4.5 Rest phase

Finally, a resting period was inserted such that the duration of each trial was 25 s. The duration of this phase varied from 1 to 7 s.

#### 2.2.2 Luminance task

The luminance task was conducted to confirm the ability to discriminate abstract objects by comparing them with their own criteria. Twenty-eight rectangular stimuli were prepared with a gray color whose RGB value was between 95 and 230 and placed every 5 steps. Participants rated the subjective brightness of the gray color within 4 s after the stimulus was shown for 3 s. During the inter-trial time for 3 s, fixation was shown on the screen. Stimuli were shown on a black background. Each stimulus was shown once in a pseudo-random order. The 10 s rest was added before the first trial and after the last trial; thus, the total task time was 300 s (28×10 + 20 s for rest).

### 2.3 Data acquisition

A 3T whole-body scanner (Discovery MR750; GE Medical Systems, Milwaukee, WI, USA) with a 32-element phased-array head coil was used. Functional volumes were acquired using T2*-weighted gradient-echo-planar imaging (EPI) sequences (40 oblique slices, 3.0 mm in thickness with a 0.5 mm slice gap, repetition time (TR) = 2500 ms, flip angle (FA) = 80°, echo time (TE) = 25 ms, field of view (FOV) = 192 × 192 mm, digital in-plane resolution = 64 × 64 pixels, and pixel dimension = 3 × 3 mm). Axial slices were sequentially acquired in ascending order. A high-resolution anatomical T1-weighted image was also acquired by a fast-spoiled gradient recalled imaging sequence (TR = 6.31 ms; TE = 1.94 ms; FA = 11°; 256 × 256 matrix; 196 slices; voxel dimensions = 1 × 1 × 1 mm).

### 2.4 Functional magnetic resonance imaging data processing

#### 2.4.1 Preprocessing

The imaging data were first preprocessed using the Multivariate Exploratory Linear Optimized Decomposition into Independent Components (MELODIC) of the FMRIB Software Library (FSL) software (http://www.fmrib.ox.ac.uk/fsl/). Preprocessing consisted of: 1) rigid-body head-motion correction using the motion correction FMRIB linear image registration tool, known as MCFLIRT, 2) regular up slice timing correction, 3) Brain Extraction Tool (BET) brain extraction, 4) spatial smoothing using a kernel of full width at half maximum at 4 mm, and 5) high-pass temporal filtering (cutoff = 100 s). Then, since head motion can affect fMRI results, rigid artifact removal was conducted with FMRIB's independent component analysis [ICA]-based Xnoiseifier (FIX) tool (Salimi-Khorshidi et al., 2014) choosing a conservative threshold of ’20’ to reduce the risk of removing signal components; this threshold determines the binary classification of any given component. Bad ICA components (such as movement-related components, white matter fluctuations, susceptibility-related artifacts, cardiac pulsation, major veins, etc.) based on spatial and temporal features were manually identified via FSL's MELODIC ICA tool by a researcher (M.S. and T.K.) following Griffanti et al. (2017).

After denoising the data, the following processing and statistical analyses were performed using the Statistical Parametric Mapping (SPM12) package (Welcome Department of Imaging Neuroscience, London, UK). Each participant's T1-weighted anatomical image was co-registered with the image representing the mean of all EPI images for each participant. The co-registered anatomical image was processed using a unified segmentation procedure combining segmentation, bias correction, and spatial normalization (Ashburner and Friston, 2005); the same normalization parameters were then used to normalize the EPI images.

#### 2.4.2 Statistical analysis

Behavioral data and parameter estimates for regions of interest (ROIs) were analyzed using the SPSS software. In the *post-hoc* analyses, which examined significant simple main effects, we used pairwise comparisons for the estimated marginal means, for both between- and within-subject factors, with p values being corrected to control for multiple tests using the Bonferroni correction.

##### 2.4.2.1 Initial individual analysis

After preprocessing, task-related activation was evaluated using a general linear model (Friston et al., 1994; Worsley and Friston, 1995). The design matrix contained regressors of 3 fMRI runs. Each run included 6 regressors of interest (2 Speakers × 3 Listener's Responses) that were modeled at the onsets of the listener's responses. The duration of each regressor was 3.3 s, corresponding to the duration of the auditory response (Fig. 1). Additionally, each run included the following 5 regressors: 1 for the Preparation phase, 2 for the Speaker's Action phase (SELF or PC), 1 for the Rating phase, and 1 for the button press. The blood-oxygen-level dependent signal for all the tasks was modeled with boxcar functions convolved with a canonical hemodynamic response function characterized by 2 gamma functions: 1 modeling the peak and 1 modeling the undershoot. Six regressors of rigid-body head motion parameters (3 displacements and 3 rotations) were included as regressors of no interest. A high-pass filter with a cutoff of 128 s was also applied to remove low-frequency signal components. Assuming a first-order autoregressive model, the serial autocorrelation was estimated from the pooled active voxels with the restricted maximum likelihood procedure and used to whiten the data (Friston et al., 2002). No global scaling was performed. To calculate the estimated parameters, a least-squares estimation was performed on the whitened data. The weighted sum of the parameter estimates in the individual analyses served as the contrast images. The contrast images obtained from the individual analyses represented the normalized task-related increments of each participant's MR signal.

##### 2.4.2.2 Subsequent group analysis

Contrast images from the individual analyses were used for the group analysis. A flexible factorial design was adopted to construct a single design matrix that comprised the factors of the group (ASD and TD), condition (2 × 3 task conditions in the Listener's Response phase), and the individual participant factor. Since signal loss occurred around the paranasal sinus, in regions such as the striatum and medial orbitofrontal cortex, the present investigation focused on the differential activation between TD and ASD individuals on self-related regions and conducted an ROI analysis on the arMPFC, rather than a whole-brain analysis. To avoid a circular analysis (Kriegeskorte et al., 2009), the ROI was defined independently as a 6 mm sphere from the peak of the mPFC (Montreal Neurological Institute coordinates: 4, 58, 16) based on the contrast of self-relatedness (SELF > PC) in our previous study (Sumiya et al., 2017). We averaged the parameter estimates of 6 conditions for all voxels within the ROI for each group and calculated the contrast estimate after each PC condition, serving as a baseline for the SELF conditions. The activation pattern in the arMPFC was examined by conducting a two-way analysis of variance (ANOVA; Group x Self-relatedness) and *post-hoc* pairwise comparisons of the parameter estimates using SPSS statistical software.

### 2.5 Head motion during scanning

As head motion can affect fMRI results, the motion parameters of 3 displacements (x, y, and z axes) and 3 rotations (pitch, roll, and yaw) were evaluated between the ASD and TD groups. Specifically, the difference in the maximum and minimum values of each parameter within a run and the standard deviation of the time-series values of each parameter within a run were calculated (Okamoto et al., 2018, 2017). Supplemental Table 1 shows the means of these values in the 3 runs. An independent sample *t*-test revealed no significant differences between the 2 groups in all values (all *p* > 0.2). Next, the correlations between the regressors of each condition (SELF_No, SELF_Single, SELF_Group, PC_No, PC_Single, PC_Group) and the 6 motion parameters for the 2 groups (Supplemental Table 2) were examined. An independent sample *t*-test revealed no significant differences between the 2 groups (all *p* > 0.05).

### 2.6 Data visualization

Graphs for behavioral data were prepared using the RainCloudPlots R-script (Allen et al., 2018) (https://github.com/RainCloudPlots/RainCloudPlots); these provide a combination of the box, violin, and dataset plots. In the dataset plot, each dot represents a respective data point. Graphs for neural data were prepared using GraphPad PRISM 7 (GraphPad Software, San Diego, CA, USA).

## 3 Results

### 3.1 Behavioral results

#### 3.1.1 The pseudo-interactive joke task

**Fig. 2:**
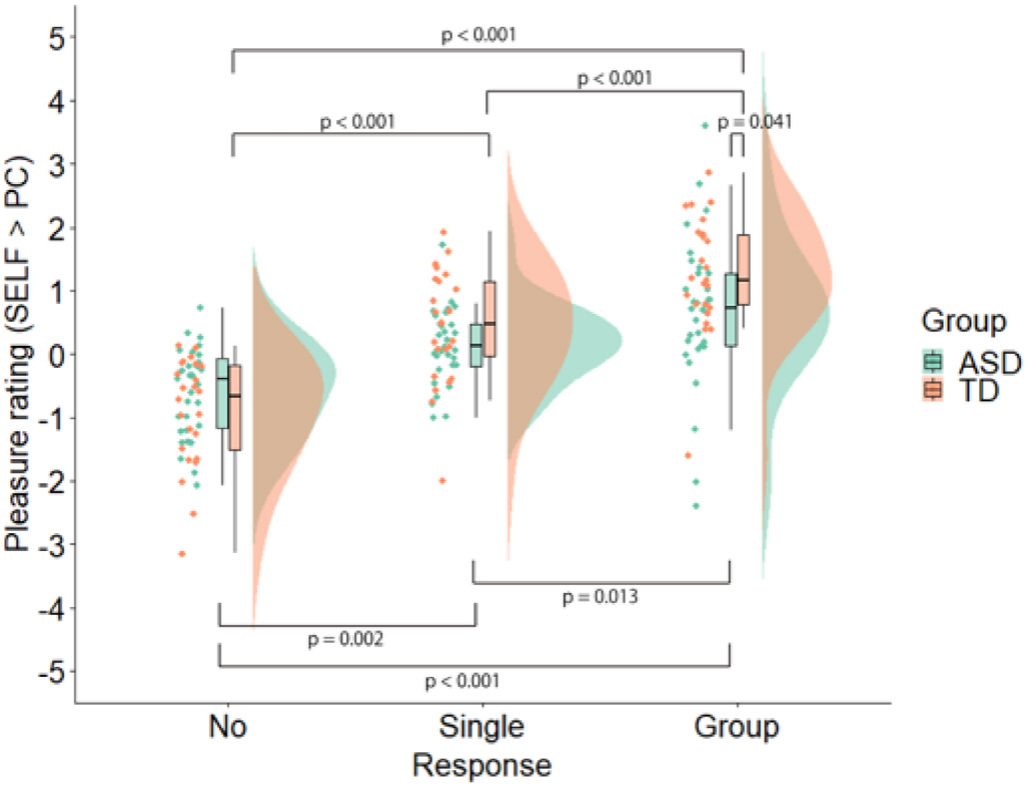
Pleasure rating.

The participants rated subjective pleasure after the listener's response in each trial. The rated subjective pleasure was compared between ASD and TD groups after each PC condition, serving as a baseline for the SELF conditions. Greater laughter contingent upon one's own action yielded greater pleasure in both ASD and TD groups, but increments were lesser in the ASD group (Fig. 2). Two-way ANOVA (2 levels of Group x 3 levels of Listener's Response) values indicate the pleasure rating, interaction (F_(1.515, 80,312__)_ = 4.223, *p* = 0.027, pη^2^ = 0.074), and the main effect of Listener's Response (F_(1.515, 80,312__)_ = 53.400, *p* < 0.001, pη^2^ = 0.502) were significant, but not the main effect of Group (F_(1,53__)_ = 1.914, *p* = 0.172, pη^2^ = 0.035). *Post-hoc* analyses revealed significant differences between each condition (No vs Single: *p* = 0.002, No vs Group: *p* < 0.001, Single vs Group: *p* = 0.013) in the ASD group and significant differences between each condition (No vs Single: *p* < 0.001, No vs Group: *p* < 0.001, Single vs Group: *p* < 0.001) in the TD group. Also, there were significant differences among ASD and TD participants in the Group laughter condition (*p* = 0.041), but not the Single laughter (*p* = 0.139) or No laughter conditions (*p* = 0.124).

The participants rated subjective pleasure after the listener's response in each trial. After setting each PC condition as the baseline for the SELF conditions, a two-way ANOVA (2 levels of Group x 3 levels of Listener's Response) was conducted. There was an significant interaction (F_(1.515, 80,312__)_ = 4.223, *p* = 0.027, pη^2^ = 0.074). ANOVA, analysis of variance; ASD, autism spectrum disorder; TD, neuro-typical development. During the SELF condition, the participant uttered the punchline of the joke while during PC condition, the participant listened to the punchline read aloud by the PC.

#### 3.1.2 The luminance task

There was no significant difference regarding the ability to discriminate abstract objects by comparing with their own criteria between people with ASD and TD individuals (Supplemental Fig. 1). The two-way ANOVA (2 levels of Group x 3 levels of Luminance) on the reported stimuli brightness revealed that there was no interaction (F_(2, 84__)_ = 0.423, *p* = 0.656, pη^2^ = 0.010). The main effect of Luminance was significant (F_(2, 84)_ = 142.499, *p* < 0.001, pη^2^ = 0.772), but the main effect of Group was not significant (F_(1, 42)_ = 3.743, *p* = 0.060, pη^2^ = 0.082). Also, there was no significant simple main effect on each level of Luminance between people with ASD and TD participants: Low (*p* = 0.104), Middle (*p* = 0.090), and High (*p* = 0.051).

### 3.2 Functional magnetic resonance imaging results

**Fig. 3:**
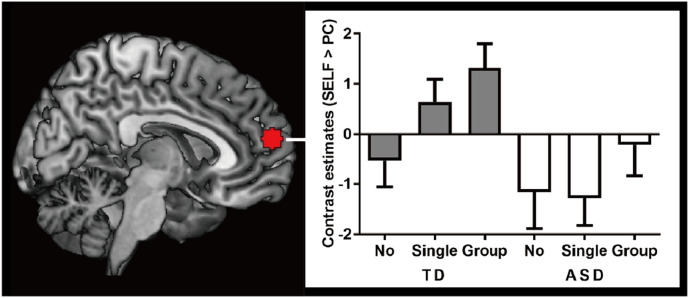
The task-related activation in the arMPFC.

To evaluate activity in the arMPFC during the Listener's Response phase, activity (parameter estimates) was extracted from independent ROIs (6 mm sphere from a peak coordinate in our previous study). After setting each PC condition as the baseline of SELF conditions, a two-way ANOVA (2 levels of Group x 3 levels of Listener's Response) was conducted. There was a significant main effect of Group (F_(1, 42__)_ = 13.230, *p* = 0.001, pη^2^ = 0.240), but no main effect of Listener's Response (F_(1.753, 73.641__)_ = 2.780, *p* = 0.075, pη^2^ = 0.062) nor interaction (F_(1.753, 73.641)_ = 0.600, *p* = 0.530, pη^2^ = 0.014) (Fig. 3). *Post-hoc* analyses revealed no significant difference between each condition (No vs Single: *p* = 1.000, No vs Group: *p* = 1.000, Single vs Group: *p* = 0.403) in the ASD group and no significant difference between each condition (No vs Single: *p* = 0.550, No vs Group: *p* = 0.140, Single vs Group: *p* = 0.890) in the TD group. Also, there were significant differences between ASD and TD in the Single laughter condition (*p* = 0.009), but not in the Group laughter (*p* = 0.057) or No laughter conditions (*p* = 0.471).

A region of interest (ROI), shown on the left, was placed on the anterior rostral medial prefrontal cortex (6 mm sphere from a top coordinate of our previous study (MNI coordinates: 4, 58, 16)). After setting the PC conditions as the baseline of the SELF conditions, a two-way ANOVA (2 levels of Group x 3 levels of Listener's Response) was performed on the contrast estimates (of an arbitrary unit). There was an significant main effect of Group (F_(1, 42__)_ = 13.230, *p* = 0.001, pη^2^ = 0.240). Data are presented as the mean ± standard error of the mean. ANOVA, analysis of variance; ASD, autism spectrum disorder; TD, neuro-typical development. During the SELF condition, the participant uttered the punchline of the joke while during PC condition, the participant listened to the punchline read aloud by the PC.

## 4 Discussion

### 4.1 Pleasantness during social interaction

People with ASD reported less pleasure from other's responses contingent on their actions than TD individuals. Misunderstanding the task procedure was unlikely because ASD participants successfully rated the subjective brightness of the objects similar to TD participants. Furthermore, individuals with ASD reported significant pleasure in Group laughter compared to the other conditions. This finding indicates that individuals with ASD feel pleasure in social action-outcome contingencies, although attenuated. Children with ASD exhibit less of a positive effect during interactions, suggesting a lower level of shared fun (Bauminger-Zviely and Agam-Ben-Artzi, 2014; Bauminger et al., 2008). On the other hand, many qualitative studies (Carrington et al., 2003; Gurbuz et al., 2019; O'Hagan and Hebron, 2017; Sumiya et al., 2018) explored the pleasurable experience regarding friendship and the desire to form and maintain friendships in certain individuals with ASD. The present finding, in accord with previous studies, indicates the need for support for ASD participants during social interaction.

### 4.2 Anterior rostral medial prefrontal cortex activation while processing self-relevant outcomes

As expected, the ROI analysis with predefined arMPFC ROIs based on the contrast of self-relevance revealed reduced arMPFC activation in ASD participants compared to TD individuals. As the arMPFC is associated with social contingency processing (Sumiya et al., 2017), this reduced activation likely represents the attenuated processing of self-relevant outcomes. Previously, Krueger et al. (2009) proposed that the MPFC represents event simulators that encompass a multi-modal representation of social event knowledge distributed throughout the brain, including the dorsal MPFC to help with inferences about the other person-schemata and the ventral MPFC for inferences about self-schemata. Consistent with this proposal, previous meta-analysis studies have shown the existence of a functional gradient along an axis from self to others within the mPFC (Denny et al., 2012; Mitchell et al., 2006). Krueger et al. (2009) also proposed that the rostral MPFC represents more general simulators that integrate information about goal knowledge (i.e., inferences about the likely action performed by the agent) with information about the outcome of one's own actions. Therefore, in the present study, TD participants may have paid attention to the listener's responses to their own actions to infer the mental state of the listener (Sumiya et al., 2017). On the other hand, such arMPFC activation was attenuated in ASD participants. This finding indicates that ASD participants may be deficient in integrating other's responses as the feedback of their own actions towards others.

Also, the present findings are consistent with previous studies that found atypical self-relevant processing in individuals with ASD (Perrykkad and Hohwy, 2019; Nijhof and Bird, 2019; Huang et al., 2017; Williams, 2010; Uddin, 2011; Cañigueral and Hamilton, 2019). For example, atypical responses of the arMPFC, a critical node of self-relevant processing (Lieberman et al., 2019; Sugiura, 2013), were reported by self-appraisal (Lombardo et al., 2010; Pfeifer et al., 2013) or self-selected picture evaluation (Kishida et al., 2019) in children and adults with ASD. In the context of social interaction, other's positive feedback associated with self-action motivates social orientation (Schilbach, 2016; Vernetti et al., 2017). Thus, the attenuated processing of self-relevance in the arMPFC impacts the way one perceives the reward value from the responses of others.

The present behavioral and neuroimaging findings suggest that people with ASD are less likely to have fun during social interaction because of attenuated self-relevant outcome processing; consequently, such deficits may dampen the motivation to interact with others. Thus, with an interactive experimental paradigm, the present study succeeded in characterizing the reward of social interaction as social contingency processing. The attenuated responses of its neural substrates in ASD participants support the social motivation hypothesis of ASD.

Notably, the hypo-activation of the arMPFC associated with self-relevant outcome monitoring does not mean that individuals with ASD cannot obtain rewards through social interaction. Instead, the relative preference for social interaction is weak in ASD participants due to difficulties in self-relevant processing. As impairments in self-relevant processing are associated with challenges in social-communicative abilities (Gillespie-Smith et al., 2018), understanding the developmental changes and individual differences of self-relevant processing in the arMPFC may contribute to the exploration of pleasantness predictability during social activities within individuals with ASD and intervention. Previous intervention studies in young children with ASD show that improving joint attention initiation enhanced their self-awareness (Gulsrud et al., 2014; Murza et al., 2016), indicating a strong relationship between self-relevant processing and social interaction initiation. As the medial prefrontal cortices are related to initiating joint attention (Schilbach et al., 2010), future studies exploring the detailed neural mechanisms of initiating joint attention are warranted to determine how to enhance self-relevant processing during social interaction. Further, studies on people with ASD have shown that increment of activation around the arMPFC, during a social judgment task induced by intranasal oxytocin, lead to improvements in the social-communication difficulties (Watanabe et al., 2014; Aoki et al., 2015). These studies indicate that people with ASD may be able to better handle their social interaction by inducing intranasal oxytocin through a self-relevant increased activation of arMPFC.

### 4.3 Limitation

This study has a few limitations. The first limitation is about the type of feedback stimuli. Only social stimuli without other non-social rewards were used. Recent systematic reviews and meta-analyses regarding the social motivation hypothesis of ASD (Bottini, 2018; Clements et al., 2018) expanded the hypothesis by finding that differential reward processing on ASD is not only social but also includes non-social stimuli and restricted interests. Hence, these studies propose that general atypical reward processing encompasses social rewards, non-social rewards, and restricted interests (Bottini, 2018; Clements et al., 2018). Similarities and differences between the processing of social and non-social stimuli need to be explored in future studies.

Another limitation is that the manner in which neural reward processing on social action-outcome contingencies is different between individuals with ASD and TD people is still unclear. In this study, we found different social reward processing in behavior between individuals with ASD and TD, but not at the neural level. Previously, social reward processing was shown to be represented by the functional connectivity between the arMPFC and other regions, such as the striatal reward and perceptual areas (Sumiya et al., 2017). However, in this study, whole-brain or functional connectivity analyses could not be conducted because of a technical difficulty: a huge signal loss occurred around the paranasal sinus, including the striatum and medial orbitofrontal cortex. Therefore, in our future studies, to obtain an increased understanding of social reward processing in people with ASD, we will focus on and investigate functional connectivity during social interaction.

## 5 Conclusion

Using a social contingency task, less pleasure contingent on other's responses to one's actions was observed in individuals with ASD, associated with attenuated arMPFC activation, representing self-relevant outcome processing. Thus, weak self-relevant information processing dampens the rewarding nature of social interaction for people with ASD.

## Funding

This work was supported by a KAKENHIgrant (17H07336) to MS, a KAKENHIgrant (17K17766) to YO, a KAKENHIgrant (15H05875) to TK, and a KAKENHIgrant (15H01846) to NS from the Japan Society for the Promotion of Science. This research is partially supported by the Strategic Research Program for Brain Sciences from the Japan Agency for Medical Research and Developmentunder grant numbers JP18dm0107152 and JP18dm0307005.

## CRediT authorship contribution statement

**Motofumi Sumiya:** Conceptualization, Data curation, Formal analysis, Funding acquisition, Investigation, Methodology, Software, Validation, Visualization, Writing - original draft. **Yuko Okamoto:** Data curation, Funding acquisition, Investigation, Resources, Software. **Takahiko Koike:** Conceptualization, Formal analysis, Funding acquisition, Methodology, Software. **Tsubasa Tanigawa:** Data curation, Investigation. **Hidehiko Okazawa:** Conceptualization, Resources, Software. **Hirotaka Kosaka:** Conceptualization, Data curation, Funding acquisition, Investigation, Project administration, Resources, Software, Supervision. **Norihiro Sadato:** Conceptualization, Funding acquisition, Project administration, Software, Supervision, Writing - original draft.

## Declaration of competing interest

The authors declare no competing financial interests.

## Appendix Supplementary materials

Image, application 1
