# Supplementary material for: Attenuated activation of the anterior rostral medial prefrontal cortex on self-relevant social reward processing in individuals with autism spectrum disorder
Source: Neuroimage Clin. 2020 Mar 19;26:102249. doi: 10.1016/j.nicl.2020.102249 (PMC7176988; doi:10.1016/j.nicl.2020.102249)
Supplement: Supplementary file 1 [file mmc1.docx]

**Supplementary Information**

**Attenuated activation of the anterior rostral medial prefrontal cortex on self-relevant social reward processing in individuals with autism spectrum disorder**

Motofumi Sumiya^1,2,3^, Yuko Okamoto^4,5,6^, Takahiko Koike^1^, Tsubasa Tanigawa^1^, Hidehiko Okazawa^7^, Hirotaka Kosaka^4,8^, Norihiro Sadato^1,*^

1: Division of Cerebral Integration, National Institute for Physiological Sciences, Aichi, Japan

2: Research Fellow of the Japan Society for the Promotion of Science, Tokyo, Japan

3: Graduate School of Informatics, Nagoya University, Aichi, Japan

4: Research Center for Child Mental Development, University of Fukui, Fukui, Japan

5: ATR-Promotions, Brain Activity Imaging Center, Kyoto, Japan

6: Advanced Telecommunications Research Institute International, Kyoto, Japan

7: Biomedical Imaging Research Center, University of Fukui, Fukui, Japan

8: Department of Neuropsychiatry, University of Fukui, Fukui, Japan

*Corresponding author details

Norihiro Sadato, MD, PhD, Email: sadato@nips.ac.jp

Division of Cerebral Integration, National Institute for Physiological Sciences, Okazaki, 444-8585, Japan Tel: +81-564-55-7841; Fax: +81-564-55-7843;

**This information includes:**

1 Supplemental Figure

2 Supplemental Tables


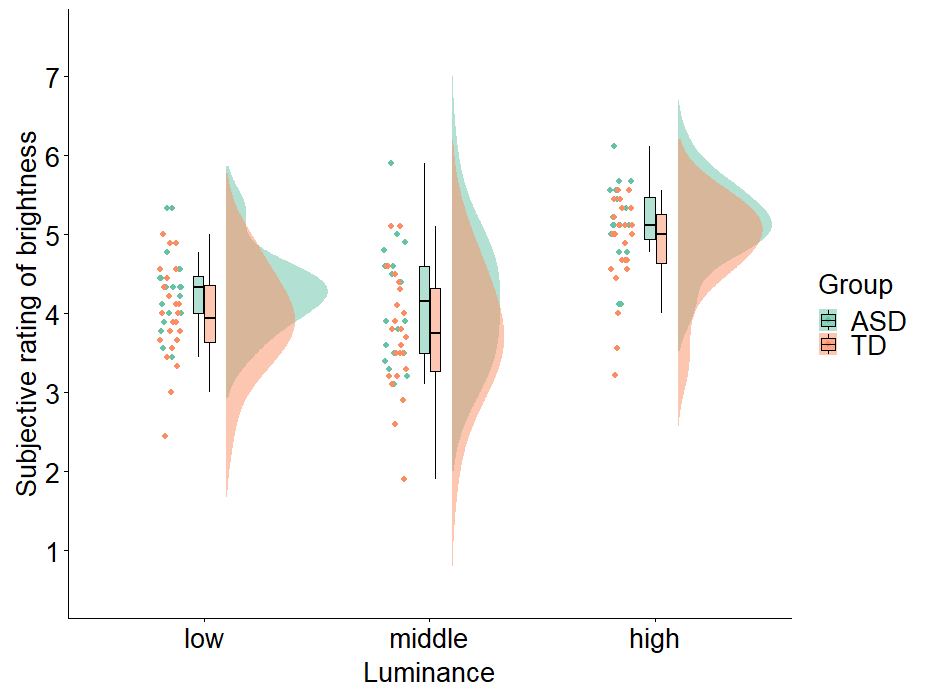


**Supplemental Figure 1. Luminance task**

ASD, autism spectrum disorder; TD, neuro-typical development; ANOVA, analysis of variance. The luminance task was conducted to confirm the participants’ ability to discriminate abstract objects by comparing them with their own criteria. A two-way ANOVA (2 level of Group x 3 level of Luminance) on the reported stimuli brightness indicated there was no interaction (F_(2, 84)_ = 0.423, p = 0.656, pη^2^ = 0.010).

**Supplemental Table 1. Motion parameters of the ASD and TD groups**

|  | ASD | | |  | TD | | |
| --- | --- | --- | --- | --- | --- | --- | --- |
| Mean of the maximum amplitude within a run | | | | | | | |
| Translation |  |  |  |  |  |  |  |
| x | 0.022 | ± | 0.011 |  | 0.022 | ± | 0.012 |
| y | 0.009 | ± | 0.005 |  | 0.008 | ± | 0.004 |
| z | 0.007 | ± | 0.005 |  | 0.006 | ± | 0.002 |
| Rotation |  |  |  |  |  |  |  |
| x | 0.414 | ± | 0.279 |  | 0.337 | ± | 0.146 |
| y | 0.464 | ± | 0.256 |  | 0.414 | ± | 0.197 |
| z | 1.541 | ± | 0.795 |  | 1.466 | ± | 0.979 |
|  |  |  |  |  |  |  |  |
| Mean of the standard deviation of variations within a run | | | | | | | |
| Translation |  |  |  |  |  |  |  |
| x | 0.004 | ± | 0.002 |  | 0.004 | ± | 0.002 |
| y | 0.002 | ± | 0.001 |  | 0.002 | ± | 0.001 |
| z | 0.001 | ± | 0.001 |  | 0.001 | ± | 0.001 |
| Rotation |  |  |  |  |  |  |  |
| x | 0.084 | ± | 0.052 |  | 0.075 | ± | 0.036 |
| y | 0.089 | ± | 0.053 |  | 0.082 | ± | 0.037 |
| z | 0.309 | ± | 0.170 |  | 0.293 | ± | 0.198 |

ASD, autism spectrum disorders; TD, neuro-typical development. Data are presented as the mean ± the standard deviation. The translation is shown in mm and the rotation is shown in radians. Note that no parameters showed significant differences between the ASD and TD groups.

**Supplemental Table 2. Correlation of motion parameters and regressors of each condition**

|  | SELF_No | | |  | SELF_Single | | |  | SELF_Group | | |
| --- | --- | --- | --- | --- | --- | --- | --- | --- | --- | --- | --- |
| ASD |  |  |  |  |  |  |  |  |  |  |  |
| Translation | | | | | | | | | | | |
| x | -0.009 | ± | 0.059 |  | -0.009 | ± | 0.059 |  | -0.009 | ± | 0.059 |
| y | -0.014 | ± | 0.043 |  | -0.014 | ± | 0.043 |  | -0.014 | ± | 0.043 |
| z | -0.003 | ± | 0.072 |  | -0.003 | ± | 0.072 |  | -0.003 | ± | 0.072 |
| Rotation | | | | | | | | | | | |
| Pitch | 0.011 | ± | 0.060 |  | 0.011 | ± | 0.060 |  | 0.011 | ± | 0.060 |
| Roll | -0.026 | ± | 0.045 |  | -0.026 | ± | 0.045 |  | -0.026 | ± | 0.045 |
| Yaw | 0.009 | ± | 0.053 |  | 0.009 | ± | 0.053 |  | 0.009 | ± | 0.053 |
| TD |  |  |  |  |  |  |  |  |  |  |  |
| Translation | | | | | | | | | | | |
| x | 0.012 | ± | 0.052 |  | 0.012 | ± | 0.052 |  | 0.012 | ± | 0.052 |
| y | -0.005 | ± | 0.044 |  | -0.005 | ± | 0.044 |  | -0.005 | ± | 0.044 |
| z | 0.004 | ± | 0.043 |  | 0.004 | ± | 0.043 |  | 0.004 | ± | 0.043 |
| Rotation | |  |  |  |  |  |  |  |  |  |  |
| Pitch | 0.006 | ± | 0.057 |  | 0.006 | ± | 0.057 |  | 0.006 | ± | 0.057 |
| Roll | -0.017 | ± | 0.048 |  | -0.017 | ± | 0.048 |  | -0.017 | ± | 0.048 |
| Yaw | 0.010 | ± | 0.053 |  | 0.010 | ± | 0.053 |  | 0.010 | ± | 0.053 |
|  | PC_No | | |  | PC_Single | | |  | PC_Group | | |
| ASD |  |  |  |  |  |  |  |  |  |  |  |
| Translation | | | | | | | | | | | |
| x | -0.009 | ± | 0.059 |  | -0.009 | ± | 0.059 |  | -0.009 | ± | 0.059 |
| y | -0.014 | ± | 0.043 |  | -0.014 | ± | 0.043 |  | -0.014 | ± | 0.043 |
| z | -0.003 | ± | 0.072 |  | -0.003 | ± | 0.072 |  | -0.003 | ± | 0.072 |
| Rotation | | | | | | | | | | | |
| Pitch | 0.011 | ± | 0.060 |  | 0.011 | ± | 0.060 |  | 0.011 | ± | 0.060 |
| Roll | -0.026 | ± | 0.045 |  | -0.026 | ± | 0.045 |  | -0.026 | ± | 0.045 |
| Yaw | 0.009 | ± | 0.053 |  | 0.009 | ± | 0.053 |  | 0.009 | ± | 0.053 |
| TD |  |  |  |  |  |  |  |  |  |  |  |
| Translation | | | | | | | | | | | |
| x | 0.012 | ± | 0.052 |  | 0.012 | ± | 0.052 |  | 0.012 | ± | 0.052 |
| y | -0.005 | ± | 0.044 |  | -0.005 | ± | 0.044 |  | -0.005 | ± | 0.044 |
| z | 0.004 | ± | 0.043 |  | 0.004 | ± | 0.043 |  | 0.004 | ± | 0.043 |
| Rotation | | | | | | | | | | | |
| Pitch | 0.006 | ± | 0.057 |  | 0.006 | ± | 0.057 |  | 0.006 | ± | 0.057 |
| Roll | -0.017 | ± | 0.048 |  | -0.017 | ± | 0.048 |  | -0.017 | ± | 0.048 |
| Yaw | 0.010 | ± | 0.053 |  | 0.010 | ± | 0.053 |  | 0.010 | ± | 0.053 |

ASD, autism spectrum disorders; TD, neuro-typical development; SELF_No, self-uttered joke without laughter as a response; SELF_Single, self-uttered joke with a single laugh; SELF_Group, self-uttered joke with group laughter; PC_No, joke uttered by a personal computer without laughter as a response; PC_Single, joke uttered by a personal computer with a single laugh as a response; PC_Group, joke uttered by a personal computer with group laughter as a response. Data are the mean ± the standard deviation. The translation is shown in mm and the rotation is shown in radians. Note that no parameters showed significant differences between the ASD and TD groups.
